# Long-Term Mortality After TAVI for Bicuspid vs. Tricuspid Aortic Stenosis: A Propensity-Matched Multicentre Cohort Study

**DOI:** 10.3389/fcvm.2022.894497

**Published:** 2022-06-21

**Authors:** Aleksandra Gasecka, Michał Walczewski, Adam Witkowski, Maciej Dabrowski, Zenon Huczek, Radosław Wilimski, Andrzej Ochała, Radosław Parma, Piotr Scisło, Bartosz Rymuza, Karol Zbroński, Piotr Szwed, Marek Grygier, Anna Olasińska-Wiśniewska, Dariusz Jagielak, Radosław Targoński, Grzegorz Opolski, Janusz Kochman

**Affiliations:** ^1^1st Chair and Department of Cardiology, Medical University of Warsaw, Warsaw, Poland; ^2^Department of Interventional Cardiology and Angiology, Institute of Cardiology, Warsaw, Poland; ^3^Division of Cardiology and Structural Heart Diseases, Medical University of Silesia, Katowice, Poland; ^4^Department of Cardiac Surgery and Transplantology, Poznan University of Medical Sciences, Poznan, Poland; ^5^Department of Cardiac and Vascular Surgery, Medical University of Gdansk, Gdansk, Poland

**Keywords:** aortic stenosis (AS), bicuspid aortic valve (BAV), transcatheter aortic valve implantation (TAVI), mortality, outcomes

## Abstract

**Objectives:**

Patients with bicuspid aortic valve (BAV) stenosis were excluded from the pivotal trials of transcatheter aortic valve implantation (TAVI). We compared the in-hospital and long-term outcomes between patients undergoing TAVI for bicuspid and tricuspid aortic valve (TAV) stenosis.

**Methods:**

We performed a retrospective registry-based analysis on patients who underwent TAVI for BAV and TAV at five different centers between January 2009 and August 2017. The primary outcome was long-term all-cause mortality. Secondary outcomes were in-hospital mortality, procedural complications, and valve performance.

**Results:**

Of 1,451 consecutive patients who underwent TAVI, two propensity-matched cohorts consisting of 130 patients with BAV and 390 patients with TAV were analyzed. All-cause mortality was comparable in both groups up to 10 years following TAVI (*HR* 1.09, 95% *CI*: 0.77–1.51). Device success and in-hospital mortality were comparable between the groups (96 vs. 95%, *p* = 0.554 and 2.3 vs. 2.1%, *p* = 0.863, respectively). Incidence of procedural complications was similar in both groups, with a trend toward a higher rate of stroke in patients with BAV (5 vs. 2%, *p* = 0.078). Incidence of moderate or severe paravalvular leak (PVL) at discharge was comparable in both groups (2 vs. 2%, *p* = 0.846). Among patients with BAV, all-cause mortality was similar in self-expanding and balloon-expandable prostheses (*HR* 1.02, 95% *CI*: 0.52–1.99) and lower in new-generation devices compared to old-generation valves (*HR* 0.27, 95% CI 0.12–0.62).

**Conclusion:**

Patients who had undergone TAVI for BAV had comparable mortality to patients with TAV up to 10 years after the procedure. The device success, in-hospital mortality, procedural complications, and PVL rate were comparable between the groups. The high rate of neurological complications (5%) in patients with BAV warrants further investigation.

## Introduction

Bicuspid aortic valve (BAV) is the most common congenital anomaly in adults, present in 1–2% of the population (1). BAV is associated with accelerated aortic valve degeneration, thoracic aorta dilation, aorta coarctation, and increased risk of infective endocarditis (2–5). Hence, patients with BAV may require aortic valve replacement at an earlier age than those with tricupid aortic valve (TAV). Transcatheter aortic valve implantation (TAVI) has become the established treatment for aortic stenosis (AS) in patients at increased risk of surgery, expanding to intermediate- and low-risk patients (6–8). As bicuspid anatomy has been considered a relative contraindication to TAVI, patients with BAV have been excluded from the hitherto randomized clinical trials (7, 8). The main concerns of TAVI in BAV patients comprised the higher risk of malposition and underexpansion of the device, resulting in significant paravalvular leak (PVL) due to heavy calcification, increased risk for aortic root rupture, coronary occlusion, and faster degeneration of bioprosthesis (9). Using current-generation devices, procedural and 1-year outcomes seem to be comparable following TAVI for bicuspid and tricuspid aortic valve disease, suggesting that TAVI is a viable treatment option for patients with BAV (10, 11). However, the long-term observations after TAVI in BAV patients are not yet available. Considering that TAVI is expanding to the younger and more healthy patients, the long-term observation of TAVI in BAV is of paramount importance (12). The goal of this study was to compare the in-hospital and long-term clinical outcomes between patients undergoing TAVI for bicuspid and tricuspid AS, and compare outcomes between self-expanding vs. balloon-expandable TAVI prostheses and between old- and new-generation devices in BAV patients.

## Methods

We conducted a multicentre registry-based analysis of patients undergoing TAVI at five experienced academic centers in Poland. The study was formally deemed exempt from Bioethical Medical Committee of Warsaw approval. The study population comprised patients with symptomatic severe AS of the bicuspid or tricuspid valve, who were qualified for TAVI by the local, interdisciplinary Heart Teams comprising a general cardiologist, an interventional cardiologist, and a cardiac surgeon (6). The primary imaging modality for the determination of aortic valve morphology was transoesophageal echocardiography until the year 2013, and multi-slice computed tomography (MSCT) from the year 2014. We excluded all patients with aborted procedures, previous AV replacement, and other valve morphologies (unicuspid, quadricuspid, or uncertain). Participating centers used standardized definitions to collect clinical information such as patient demographics, comorbidities, laboratory data, procedural details, and in-hospital outcomes. Data regarding long-term mortality were obtained from the Polish National Health Service database.

### Outcomes

The primary outcome was long-term all-cause mortality after TAVI for BAV compared to TAV. Secondary outcomes included (i) in-hospital mortality, (ii) incidence of procedural complications (life-threatening or disabling bleeding, major vascular complications, stroke, and new pacemaker implantation), and (iii) valve performance evaluated by the in-hospital echocardiography (mean and peak prosthetic valve gradients, PVL type 3 or 4). The exploratory outcomes included a comparison between self-expanding vs. balloon-expandable TAVI prostheses, as well as between old- and new-generation devices in BAV patients. All adverse outcomes were defined using Valve Academic Research Consortium-2 (VARC-2) definitions.

### Statistical Analysis

Statistical analysis was conducted using IBM SPSS Statistics, version 27.0 (IBM). Categorical variables were presented as numbers and percentages and compared using Chi-square or Fischer exact tests. A Shapiro–Wilk test was used to assess the normal distribution of continuous variables. Continuous variables were presented as mean and standard deviation (SD) or median with interquartile range (IQR) and compared using the two-sample *t*-tests or Mann–Whitney U tests. The long-term mortality rates were presented using Kaplan–Meier curves and compared using the log-rank test. It was anticipated that patients with bicuspid and tricuspid AS would have significantly different baselines and procedural characteristics. To avoid confounding due to these differences, propensity score-based matching was used. Propensity scores were calculated using a logistic regression model based on nine relevant baseline patient characteristics (covariates) with aortic valve type (bicuspid or tricuspid aortic stenosis) as the dependent variable. The covariates were age, sex (male), EuroSCORE II, peripheral artery disease, hemoglobin level, estimated glomerular filtration rate, left ventricular ejection fraction (LVEF), access site, and valve size. Missing baseline values were imputed using the Markov Chain Monte Carlo method prior to modeling. The missing procedural outcomes and follow-up data were not imputed. Patients with bicuspid AS were matched in a 1:3 ratio to those with tricuspid AS with a caliper of 0.1, producing two patient cohorts. The results are presented as hazard ratios (*HR*) and with a 95% confidence interval (*CI*). All *p*-values are two-sided, and *p* < 0.05 was considered significant for all tests.

## Results

### Baseline Characteristics

**Figure 1:**
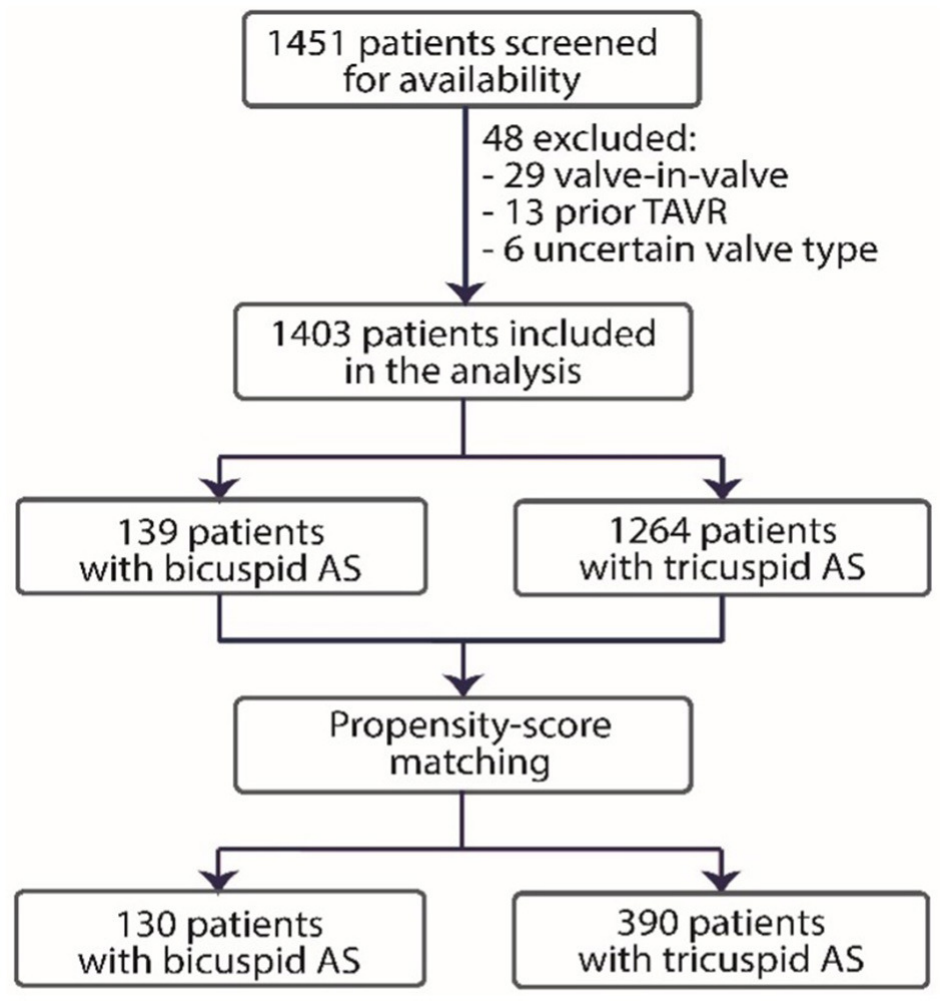
Study flowchart. TAVR, transcatheter aortic valve replacement; AS, aortic stenosis.

Between January 2009 and August 2017, a total of 1,451 patients underwent TAVI at five participating centers. Aortic valve morphology was determined based on transoesophageal echocardiography in 183 patients, including 34 patients with BAV (35% of the study population), and based on MSCT in 337 patients, including 96 patients with BAV (65% of the study population). The follow-up ended on 30 August 2020. A total of 1,403 patients (139 patients with BAV and 1,264 patients with TAV) were included in the present analysis, producing propensity-matched groups of 130 patients with BAV and 390 patients with TAV (Figure 1).

In the unmatched cohort, patients with BAV were younger (median age 79 years, IQR 73–83 years vs. 81 years, IQR76–84 years; *p* = 0.002); they had a lower EuroSCORE II-predicted risk of mortality (3.5%, IQR 2.5–5.2 vs. 4.1%, IQR 2.7–6.8%; *p* = 0.04) and fewer comorbidities. After adjusting with propensity-score matching, baseline characteristics were not significantly different (Table 1). The median procedure dates in the matched cohort were 12 November 2014 for the bicuspid AS cohort and 1 September 2014 for the tricuspid AS cohort.

**Table 1:** Baseline characteristics before and after propensity score matching.

	**Before PS matching**			**After PS matching**		
**Variable**	**TAV (*n* = 1,264)**	**BAV (*n* = 139)**	***p***	**TAV (*n* = 390)**	**BAV (*n* = 130)**	***p***
**Baseline characteristics**						
Age (years),	81 (76–84)	79 (73–83)	0.002	80 (76–84)	79 (74–82)	0.136
Gender (male)	692 (55%)	82 (59%)	0.369	198 (51%)	78 (60%)	0.068
BMI (kg/m^2^)	26.8 (24.0–30.1)	27.05 (23.9–30.00)	0.690	26.4 (23.6–30.1)	27.0 (24.0–30.0)	0.477
**Co-morbidities**						
Hypertension	868 (69%)	90 (65%)	0.346	270 (69%)	81 (62%)	0.144
Diabetes mellitus	445 (35%)	40 (29%)	0.130	108 (28%)	45 (35%)	0.134
Prior stroke/ TIA	145 (11%)	16 (12%)	0.989	63 (16%)	15 (12%)	0.202
Coronary artery disease	754 (60%)	78 (56%)	0.420	219 (56%)	75 (58%)	0.094
Myocardial infarction within the last 90 days	38 (3%)	1 (1%)	0.120	8 (2%)	1 (1%)	0.332
Prior cardiac surgery	256 (20%)	27 (19%)	0.817	52 (13%)	24 (18%)	0.152
Peripheral artery disease	343 (27%)	24 (17%)	0.012	86 (22%)	24 (18%)	0.385
Prior pacemaker	203 (16%)	16 (12%)	0.161	60 (15%)	16 (12%)	0.739
COPD	233 (18%)	25 (18%)	0.897	69 (18%)	31 (24%)	0.123
Pulmonary hypertension	175 (14%)	19 (14%)	0.955	46 (12%)	19 (15%)	0.399
Heart failure (NYHA III/IV)	983 (78%)	112 (81%)	0.448	300 (77%)	105 (81%)	0.360
EuroSCORE II (%)	4.1% (2.7–6.8%)	3.5% (2.5–5.2%)	0.040	3.8% (2.8–6.5%)	3.6% (2.6–5.1%)	0.171
**Laboratory data**						
Hemoglobin, g/dL	12.0 (10.3–13.2)	12.7 (11.0–13.6)	0.003	12.3 (11.2–13.4)	12.7 (11.0–13.6	0.761
Creatinine, mg/dL	1.1 (0.9–1.4)	1.1 (0.9–1.3)	0.765	1.2 (1.0–1.5)	1.1 (0.9–1.3)	0.213
Estimated GFR, mL/min/1.73 m^2^	55 (43–65)	58 (47–73)	0.020	56 (40–65)	57 (47–74)	0.101
**Echocardiography before TAVI**						
Ejection fraction, %	55 (47–60)	55 (43–60)	0.113	55 (40–64)	55 (41–60)	0.193
Mitral insufficiency (moderate/severe)	228 (18%)	43 (31%)	0.001	96 (25%)	31 (24%)	0.885
Tricuspid insufficiency (moderate/severe)	268 (21%)	32 (23%)	0.620	103 (26%)	30 (23%)	0.451

### Procedural Characteristics and In-hospital Outcomes

All patients in both cohorts completed follow-up at hospital discharge. Among the propensity-score matched patients, there were no procedural differences (Table 2). Patients with BAV received larger-size prostheses, with 31 mm prostheses more often used and 26 mm less often in the BAV group compared to the TAV group (*p* = 0.003, *p* < 0.001, respectively). Also, new-generation valves were used more often in patients with BAV (44 vs. 30%, *p* < 0.0001). There were no differences in the use of self-expanding and balloon-expandable valves.

**Table 2:** Procedural characteristics and in-hospital outcomes.

**Variable**	**TAV**	**BAV**	***p***
	**(*n* = 390)**	**(*n* = 130)**	
**Anesthesia**			
General	260 (67%)	89 (68%)	0.706
Local	130 (33%)	41 (32%)	0.706
**Access site**			
Transfemoral	320 (82%)	112 (86%)	0.280
Transapical	35 (9%)	5 (4%)	0.057
Other	35 (9%)	13 (10%)	0.726
**Prosthesis size (mm)**			
23	58 (15%)	21 (16%)	0.724
25	9 (2%)	3 (2%)	1.000
26	140 (36%)	26 (20%)	<0.001
27	5 (1%)	4 (3%)	0.174
29	158 (41%)	58 (45%)	0.411
31	19 (5%)	16 (12%)	0.003
34	1 (0.3%)	2 (1.5%)	0.094
**Valve type**			
CoreValve	144 (37%)	39 (30%)	0.152
Boston Lotus	44 (11%)	20 (15%)	0.218
EvolutR	71 (18%)	37 (28%)	0.013
Edwards Sapien	61 (16%)	2 (2%)	<0.001
Edwards Sapien XT	24 (6%)	8 (6%)	1.000
Edwards Sapien 3	46 (12%)	24 (18%)	0.054
Old generation^a^	273 (70%)	73 (56%)	<0.001
New generation^b^	117 (30%)	57 (44%)	<0.001
Self-expandable^c^	259 (66%)	96 (74%)	0.115
Balloon-expandable^d^	131 (34%)	34 (26%)	0.115
**Device success**	370 (95%)	125 (96%)	0.554
**Procedure complications**			
Post-dilatation due to PVL	90 (23%)	33 (25%)	0.592
Second valve implantation	4 (1.0%)	2 (1.5%)	0.635
Conversion to surgery	1 (0.002%)	0 (0.0%)	0.563
Annular rupture	0 (0.0%)	1 (0.01%)	0.083
In-hospital mortality	8 (2.1%)	3 (2.3%)	0.863
Life-threatening or disabling bleeding^*^	26 (7%)	7 (5%)	0.604
Major vascular complication^*^	33 (9%)	7 (5%)	0.254
Stroke	9 (2%)	7 (5%)	0.079
New pacemaker	54 (14%)	20 (15%)	0.664
**Post-TAVI echocardiography**			
Ejection fraction, %	52 (45–60)	55 (50–60)	0.101
Peak AV gradient, mm Hg	19 (14–26)	17 (12–23)	0.097
Mean AV gradient, mm Hg	10 (7–14)	9 (7–13)	0.165
Paravalvular leak type 3 or 4	7 (2%)	2 (2%)	0.846

* *According to VARC*.

a *CoreValve, Boston Lotus, Edwards Sapien, Edwards Sapien XT*.

b *EvolutR, Symetis Accurate, Edwards Sapien 3*.

c *CoreValve, Boston Lotus, EvolutR*.

d *Edwards Sapien, Edwards Sapien XT, Edwards Sapien 3*.

The device success and the in-hospital mortality were comparable between the BAV and TAV groups (96 vs. 95% and 2.3 vs. 2.1%, respectively). The incidence of procedural complications, such as life-threatening or disabling bleeding, major vascular complication and new permanent pacemaker implantation were similar in both groups. There was a trend toward a higher rate of in-hospital stroke in BAV patients (5 vs. 2%, *p* = 0.078).

### Valve Performance

At discharge, there were no differences between the peak and mean aortic valve gradients (*p* = 0.097; *p* = 0.165, respectively). The incidence of moderate or severe PVL (type 3 or 4) was comparable between the groups (2 vs. 2%, *p* = 0.846).

### Long-Term Survival

The median follow-up time was 4.6 years (IQR 3.8–5.5) in the BAV group and 4.8 years (IQR 2.9–5.9) in the TAV group (*p* = 0.51). The longest follow-up time was 10.0 years and 10.2 years in BAV and TAV groups, respectively.

**Figure 2:**
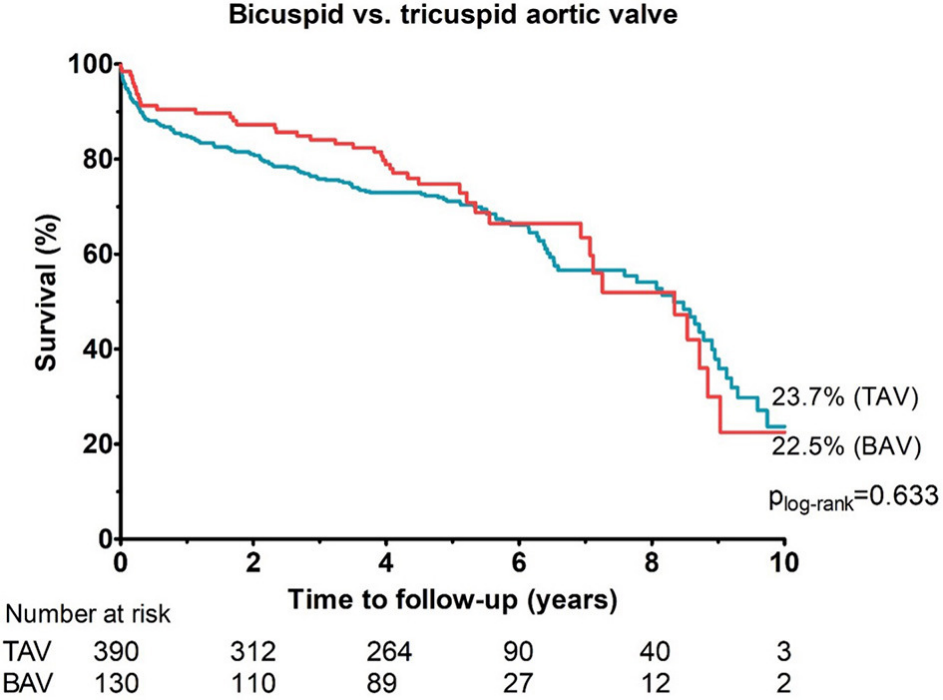
Cumulative incidences of all-cause mortality among propensity-matched cohorts with bicuspid and tricuspid aortic valve up to 10 years of follow-up.

The median survival time was 8.3 years in the BAV group and 8.3 years in the TAV group. There were no significant differences in all-cause mortality between the propensity-matched bicuspid and tricuspid AS groups detected during an observation period of up to 10 years observation period (*p*_logrank_ = 0.63;*HR* 1.09, 95% *CI*: 0.77–1.5; Figure 2).

### Comparison Between Self-Expanding vs. Balloon-Expandable Protheses in BAV Patients

**Figure 3:**
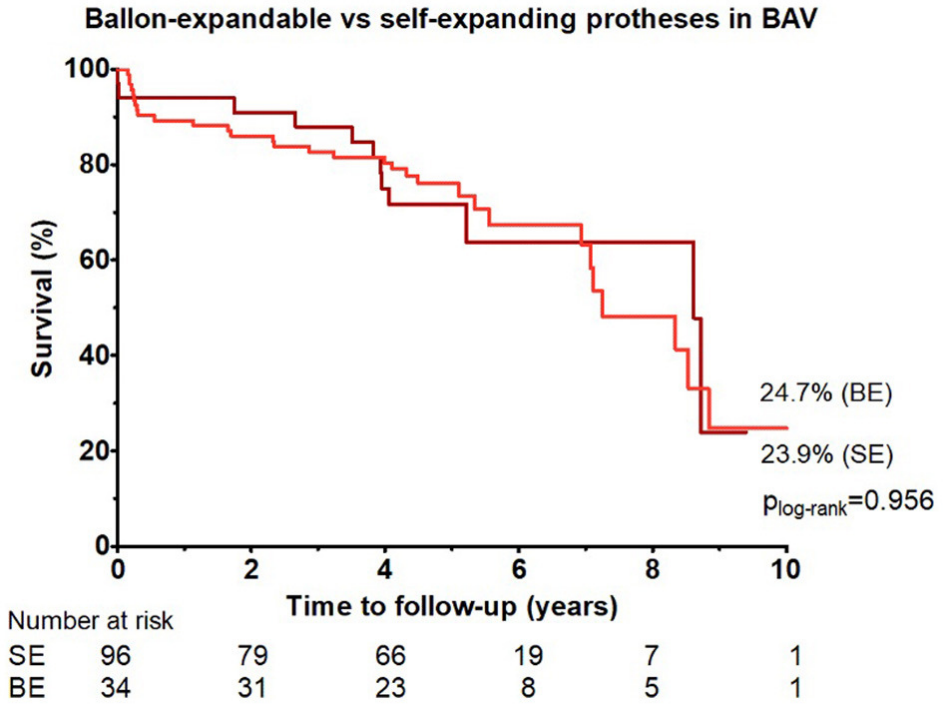
Cumulative incidences of all-cause mortality up to 10 years of follow-up among patients with bicuspid aortic valve who received balloon-expandable vs. self-expanding prostheses.

In the BAV group, 96 patients (74%) received self-expanding valve and 34 patients (26%) received balloon-expandable valve. All-cause mortality up to 10 years observation period was comparable in both groups (*p*_logrank_ = 0.956; *HR* 1.02, 95% *CI*: 0.52–1.99; Figure 3).

### Comparison Between Old-Generation and New-Generation Prostheses in BAV Patients

**Figure 4:**
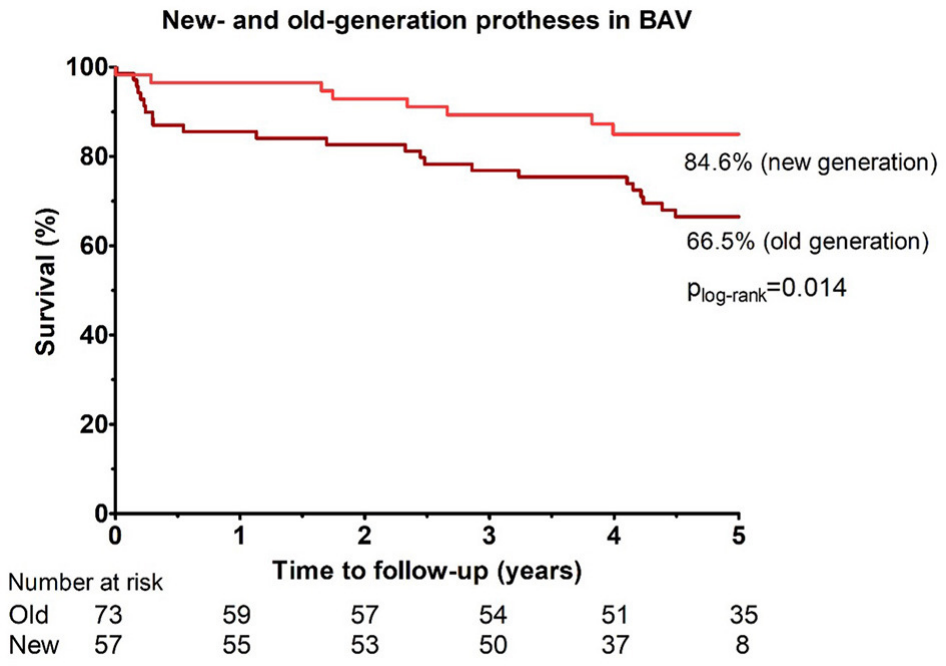
Cumulative incidences of all-cause mortality up to 5 years of follow-up among patients with bicuspid aortic valve who received new-generation vs. old-generation prostheses.

In the BAV group, 73 patients (56%) received old-generation and 57 patients (44%) received new-generation devices. The median follow-up time in patients with new-generation devices was 4.25 years (IQR 3.79–4.99 years) due to the availability of the new-generation valves on the Polish market since the year 2014. All-cause mortality up to 5 years following TAVI was substantially lower in patients who received new-generation devices compared to old-generation valves (*p*_logrank_ = 0.0016; *HR* 0.27, 95% *CI* 0.12–0.62; Figure 4).

To check whether better outcomes in the new-generation valves were due to between-group differences, we compared baseline and procedural characteristics between patients treated with the new generation and old devices in Supplementary Tables 1, 2. There were no major differences between the groups except for a higher rate of moderate/severe tricuspid insufficiency in patients treated with new-generation valves (*p* = 0.021) and a higher rate of prosthesis size 25 mm and 31 mm in patients who received old-generation devices (*p* = 0.005, *p* = 0.003, respectively).

## Discussion

This registry-based study presents the longest hitherto available follow-up in the propensity-matched patients with BAV undergoing TAVI. The main finding of our study is that patients who had undergone TAVI for bicuspid AS had comparable mortality to patients with tricuspid AS up to 10 years after the procedure, with the median follow-up time close to 5 years. The device success, rate of PVL, incidence of procedural complications and in-hospital mortality were comparable between the groups, with a trend toward the higher rate of in-hospital stroke in patients with BAV.

Patients with BAV were excluded from the pivotal trials comparing TAVI vs. surgical aortic valve replacement (SAVR) in AS. However, initial case series and registry data have shown that TAVI might be an efficient and safe alternative to SAVR in patients with BAV stenosis, with the possible caveats of increased PVL and need for permanent pacemaker implantation (13, 14). The higher risk of PVL is caused by the different anatomy of a BAV compared to a normal tricuspid structure, such as (i) asymmetry in the size of leaflets, (ii) higher point of coaptation compared to TAV, (iii) larger dimensions measured at standard anatomic points (aortic annulus, sinus of Valsalva, and ascending aorta), and (iv) higher degree and eccentricity of calcification (15). Since TAVI for bicuspid AS presents both anatomic and clinical challenges, the use of three-dimensional imaging modalities is mandatory to understand the complex and variable anatomy of BAV disease (16). Recently, it was demonstrated that the outcomes of TAVI in bicuspid AS depend on valve morphology, with the calcified raphe and excess leaflet calcification associated with increased risk of procedural complications and 1-year mortality (17). Hence, many studies focused on algorithms for valve sizing in BAV, such as attempts to compare supra-annular valve sizing with the conventional annular sizing (18) and to include the raphe length, calcium burden, and distribution in the pre-procedural evaluation of patients with BAV (19).

Although we did not evaluate the association between valve morphology and outcomes, in all patients since the year 2014 (65% of the total population and 74% of patients with BAV) the bicuspid anatomy was confirmed and valve sizing was facilitated by MSCT, which might at least partly underlie the favorable procedural outcomes. The valve performance at hospital discharge and the rate of procedural complications were similar in BAV and TAV groups. In accordance with the previous studies, there was a trend toward a higher rate of in-hospital stroke in BAV patients (11). Importantly, data presented in this analysis largely represent patients who underwent TAVI without the use of cerebral embolic protection. Routine use of embolic protection devices during TAVI has been shown to reduce the incidence of periprocedural strokes and might prove useful, especially in the BAV cohort (20).

The favorable results in our cohort were achieved despite implantation of both old- and new-generation devices. Initial studies suggested that the first-generation TAVI valves had suboptimal outcomes in patients with BAV, but later-generation valves might have outcomes similar to those seen in patients with TAV (21). In the hallmark trial with the third-generation balloon-expandable Edward Sapien 3 valve, the incidence of moderate-to-severe PVL was dramatically reduced compared to older generation valves, with the caveats of relatively high 30-day mortality rate (3.9%), new pacemaker requirement (23.5%), and asymmetrical valve deployment (38%) (22). However, recent large-scale registry-based analyses of patients treated with Edward Sapien 3 valve did not confirm the initial concerns, showing comparable rates of procedural complications in bicuspid and tricuspid AS patients, and similar 1-year rates of stroke and all-cause mortality (11, 23). Similarly, the procedural and 1-year outcomes of TAVI with new-generation self-expanding Evolut R or Evolut PRO valves were similar in patients with BAV and TAV (24). Hence, likely not only the valve generation but also other procedural advancements and improved imaging within the last years, along with the growing operator experience account for improved outcomes in patients with BAV undergoing TAVI.

In our population of intermediate-risk patients, the 2-year survival rates (80–85%) were comparable with those previously reported in the literature (82.0% for BAV vs. 83.4% for TAV) (25). The 10-year survival rate, in turn, was comparable between the bicuspid and tricuspid AS (22.5% in BAV vs. 23.7% in TAV) and higher than previously reported for tricuspid AS patients, treated with the early generation valves only (Cribier-Edwards, Edwards Sapien or CoreValve; 9.4%) (26). Likely, the improved long-term survival in our study is due to the fact that both old- and new-generation devices were used in our cohort, and in the majority of patients, MSCT was used to facilitate the procedural planning.

In our BAV cohort, all-cause mortality up to 5 years following TAVI was lower in patients who received new-generation devices, compared to those treated with old-generation valves (82.3 vs. 50.2%). A recent study that evaluated the outcomes of TAVI in 170,959 patients with bicuspid AV stenosis (3.2%) in comparison with tricuspid AV stenosis (96.7%) demonstrated comparable procedural, post-procedural, and 1-year outcomes following TAVI in both groups when current-generation devices were used (10). Better outcomes with the new-generation devices were also demonstrated in a recent meta-analyses (12, 27). Hence, TAVI seems to be a viable treatment option for patients with BAV, especially with the use of newer-generation devices and careful pre-procedural evaluation by MSCT.

Among BAV patients, there were no differences in the mortality rate up to 10 years in patients who received self-expandable vs. balloon-expanding valves (24.7, 23.9%). Recent registry-based trials and a meta-analysis of seven studies including 706 patients confirmed the feasibility of both balloon-expandable and self-expanding valve implantation in bicuspid AS, with similar rates of 30-day and 1-year mortality and stroke (12, 28). Balloon-expandable valves were associated with lower rates of new pacemaker implantation and PVL but carried a higher risk of annular rupture (12). Further randomized controlled trials are required to compare outcomes between self-expandable vs. balloon-expanding valves in BAV patients.

Our study cohort comprised intermediate-risk patients, as demonstrated by the EuroSCORE II-predicted risk of mortality (3.6 and 3.8% in the propensity-score matched patients with BAV and TAV, respectively). As such, our results suggest that TAVI may be safe and effective not only in high-risk but also intermediate-risk patients with BAV. On the other hand, the high rate of neurological complications (5%) and new pacemaker implantations (12–15%) in patients with BAV are significant drawbacks of TAVI in BAV and warrant further careful investigation. Moreover, our results are not applicable and therefore should not be extrapolated to the low-risk patients with BAV. The ongoing Low-Risk Bicuspid Study designed to evaluate the procedural safety and efficacy of TAVI in patients with BAV at low surgical risk might provide the first evidence-based data regarding the TAVI performance in low-risk BAV patients. The preliminary results of this study such as a total of 150 patients showed favorable 30-day results, with low rates of death and disabling stroke (1.3%), high device success rate (95.3%) and no moderate-to-severe PVL (29). Given that up to 50% of low-risk patients undergoing aortic valve replacement have BAV disease, the results of Low-Risk Bicuspid Study with the planned 10-year follow-up are crucial to determine the optimal interventional treatment method in these patients (29). The next step would be a randomized trial comparing TAVI to SAVR in intermediate- or low-risk BAV stenosis patients. Finally, there is a need for a prospective study with long-term follow-up such as BAV patients undergoing TAVI with new-generation devices to better understand TAVI valve durability in bicuspid anatomy (30).

## Limitations

Our study has several limitations. First, the number of patients with BAV was low and does not allow gaining strong clinical and statistical conclusions regarding TAVI in this specific subgroup. The low number of procedures and institutional, learning curve may have influenced the results and therefore the comparisons between the group. However, the number of procedures in both groups gradually increased over the years, with similar slopes on both lines (Supplementary Figure 1), implying a comparable impact of the learning curve on TAVI performance in patients with BAV and TAV. Second, there was no independent imaging core laboratory to confirm bicuspid anatomy. Third, the selection of prosthesis was at the operator's discretion, which may have affected the observed outcomes. Fourth, we did not evaluate the incidence of long-term valve performance and major cardiovascular outcomes besides mortality in our cohort. Hence, we cannot draw any conclusions regarding valve durability in BAV patients. Fifth, although propensity-score matching adjusted for the differences in baseline characteristics, it was not possible to adjust for the different degrees of aortic valve calcification. Therefore, a selection bias toward the preference of BAV patients with less calcified valves cannot be excluded. Moreover, our cohort included intermediate-risk patients, and hence our findings are not directly applicable to younger bicuspid patients. Finally, our analysis did not include an additional control group of patients with BAV treated surgically.

## Conclusion

In this preliminary, registry-based study of propensity-matched patients who had undergone TAVI for AS, patients with BAV had a similar rate of procedural complications and comparable mortality up to 10 years, compared to patients with TAV. Among BAV patients, the long-term mortality was similar in those who received balloon-expandable vs. self-expanding valves and lower in those who received new-generation valves compared to old-generation valves. However, the high rate of neurological complications and new pacemaker implantations in BAV patients warrant caution regarding TAVI in this subgroup. Further randomized trials are needed to draw firm conclusions regarding the best treatment option in patients with BAV stenosis.

## Data Availability Statement

The raw data supporting the conclusions of this article will be made available by the authors, without undue reservation.

## Ethics Statement

The studies involving human participants were reviewed and approved by Bioethical Medical Committee of Warsaw. The patients/participants provided their written informed consent to participate in this study.

## Author Contributions

AG, JK, MW, and GO: conceptualization. AG, MW, KZ, and MG: data curation. AG, MW, and JK: formal analysis. AG, MW, RW, RP, and PSc: investigation. AG, MW, AW, ZH, RW, AO, GO, and JK: methodology. AG, MW, MD, RP, PSc, and PSz: project administration. AG, MW, BR, MG, AO, and DJ: resources. BR and KZ: software. AW, ZH, AO, GO, and JK: supervision. AG, MW, RT, and DJ: validation. AG and MW: writing—original draft. JK, RT, AW, and GO: writing—review and editing. All authors have read and agreed to the published version of the manuscript.

## Conflict of Interest

AW: proctor for Medtronic. ZH: proctor for Medtronic and Abbott. RP: lecture honoraria for Edwards Lifescience. MG: lecture honoraria, proctor and advisory board member of Boston Scientific. DJ: proctor for Edwards Lifesciences. JK: lecture honoraria and proctor for Medtronic, Boston Scientific, Abbott. The remaining authors declare that the research was conducted in the absence of any commercial or financial relationships that could be construed as a potential conflict of interest.

## Publisher's Note

All claims expressed in this article are solely those of the authors and do not necessarily represent those of their affiliated organizations, or those of the publisher, the editors and the reviewers. Any product that may be evaluated in this article, or claim that may be made by its manufacturer, is not guaranteed or endorsed by the publisher.

## Abbreviations

AS: aortic stenosis
BAV: bicuspid aortic valve
LVEF: left ventricular ejection fraction
MSCT: multi-slice computed tomography
PVL: paravalvular leak
TAV: tricuspid aortic valve
TAVI: transcatheter aortic valve implantation
VARC-2: Valve Academic Research Consortium-2.
