# Supplementary material for: Long-Term Mortality After TAVI for Bicuspid vs. Tricuspid Aortic Stenosis: A Propensity-Matched Multicentre Cohort Study
Source: Front Cardiovasc Med. 2022 Jun 21;9:894497. doi: 10.3389/fcvm.2022.894497 (PMC9253589; doi:10.3389/fcvm.2022.894497)
Supplement: Supplementary file 1 [file Data_Sheet_1.docx]

Supplementary File

Figure S1. Number of transcatheter aortic valve implantation procedures per year in BAV and TAV groups.


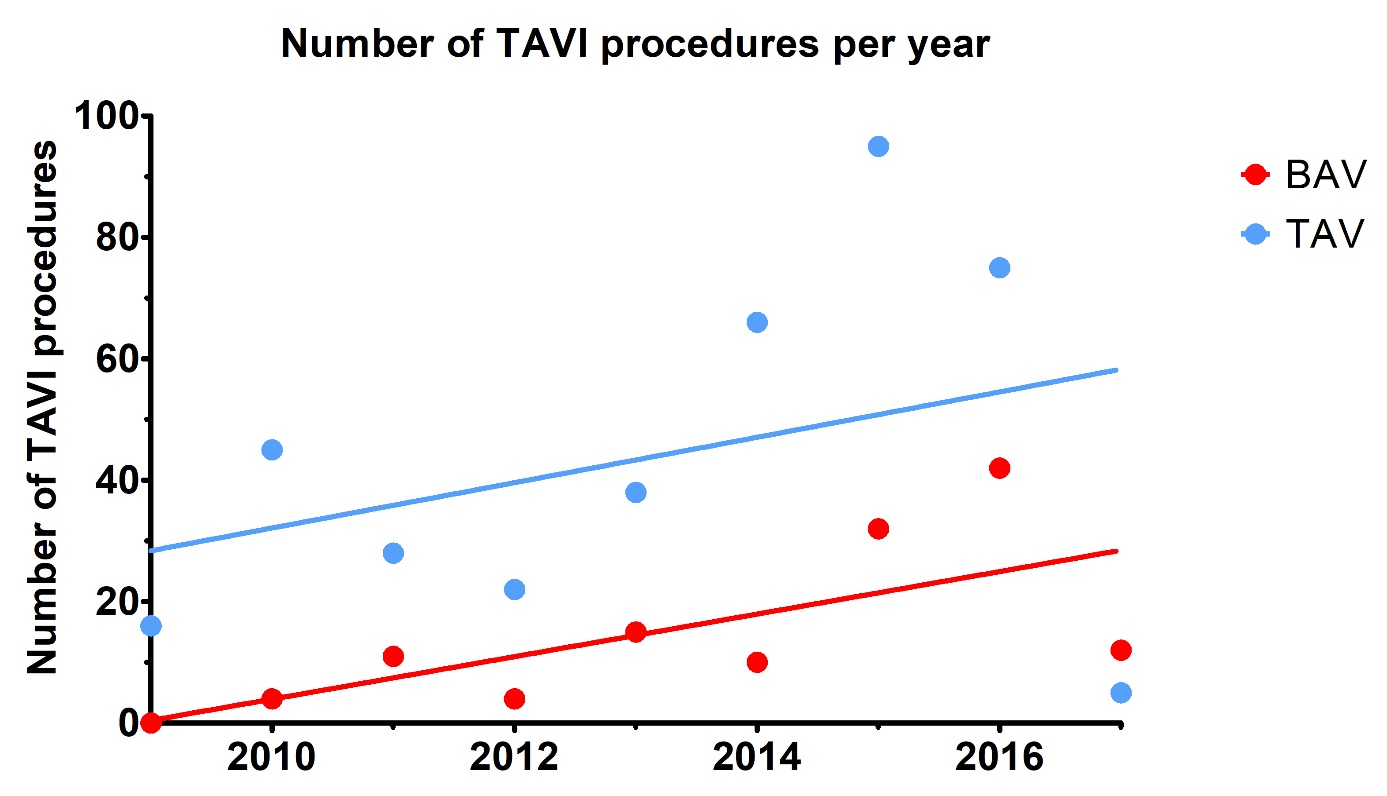


Table S1. Comparison of baseline characteristics in patients with bicuspid aortic valve treated with old generation (CoreValve, Boston Lotus, Edwards Sapien, Edwards Sapien XT) and new generation (EvolutR, Symetis Accurate, Edwards Sapien 3) prosthetic valves.

| Variable | Old generation (n=73) | New generation (n=57) | p |
| --- | --- | --- | --- |
| **Baseline characteristics** |  |  |  |
| Age (years) | 79 (72.5-83) | 79 (74.5-85) | 0.395 |
| Gender (male) | 44 (60.27%) | 34 (59.65%) | 0.943 |
| BMI (kg/m^2^) | 27.4 (23.5-30.3) | 26.70 (24.2-29.3) | 0.560 |
| **Co-morbidities** |  |  |  |
| Hypertension | 49 (67.1%) | 32 (56.1%) | 0.208 |
| Diabetes mellitus | 25 (34.25%) | 11 (19.3%) | 0.075 |
| Prior stroke/ TIA | 10 (13.7%) | 5 (8.78%) | 0.422 |
| Coronary artery disease | 45 (67.64%) | 30 (52.63%) | 0.372 |
| Myocardial infarction within the last 90 days | 1 (1.37%) | 0 (0%) | 0.375 |
| Prior cardiac surgery | 15 (20.55%) | 9 (15.79%) | 0.649 |
| Peripheral artery disease | 16 (21.92%) | 8 (14.04%) | 0.267 |
| Prior pacemaker | 10 (13.7%) | 6 (10.53%) | 0.789 |
| COPD | 14 (19.18%) | 9 (15.79%) | 0.651 |
| Pulmonary hypertension | 12 (16.44%) | 7 (12.28%) | 0.620 |
| Heart failure  (NYHA III/IV) | 59 (80.82%) | 46 (80.7%) | 0.986 |
| EuroSCORE II (%) | 13.32% (7.22-24.07%) | 9.95% (5.62-16.7%) | 0.109 |
| **Laboratory data** |  |  |  |
| Haemoglobin, g/dL | 13.0 (10.87-13.78) | 12.5 (11.2-13.3) | 0.267 |
| Creatinine, mg/dL | 1.1 (0.9-1.3) | 1.15 (0.83-1.4) | 0.580 |
| Estimated GFR, mL/min/1.73 m^2^ | 60 (47.75-74) | 53.4 (45.6-75.1) | 0.302 |
| **Echocardiography before TAVI** | |  |  |
| Ejection fraction, % | 55 (45-60) | 50 (39.5-60) | 0.385 |
| Mitral insufficiency (moderate/severe) | 14 (19.18%) | 17 (29.82%) | 0.213 |
| **Tricuspid insufficiency (moderate/severe)** | **16 (21.92%)** | **24 (42.11%)** | **0.021** |

Table S2. Comparison of procedural characteristics in patients with bicuspid aortic valve treated with old generation (CoreValve, Boston Lotus, Edwards Sapien, Edwards Sapien XT) and new generation (EvolutR, Symetis Accurate, Edwards Sapien 3) prosthetic valves.

| Variable | Old generation (n=73) | New generation (n=57) | p |  |
| --- | --- | --- | --- | --- |
| **Anaesthesia** | | | | |
| General | 52 (71.23%) | 37 (64.91%) | 0.454 |  |
| Local | 18 (24.66%) | 16 (28.07%) | 0.691 |  |
| **Access site** | | | | |
| Transfemoral | 63 (86.3%) | 49 (85.96%) | 0.956 |  |
| Transapical | 2 (2.74%) | 3 (5.26%) | 0.653 |  |
| Other | 8 (10.96%) | 5 (8.77%) | 0.774 |  |
| **Prosthesis size (mm)** | | | | |
| 23 | 10 (13.7%) | 11 (19.3%) | 0.473 |  |
| 25 | **9 (12.33%)** | **0 (0.0%)** | **0.005** |  |
| 26 | 13 (17.81%) | 13 (22.81%) | 0.514 |  |
| 27 | 3 (4.11%) | 1 (1.75%) | 0.630 |  |
| 29 | 28 (38.36%) | 30 (52.63%) | 0.113 |  |
| 31 | **10 (13.7%)** | **0 (0.0%)** | **0.003** |  |
| 34 | 0 (0.0%) | 2 (3.51%) | 0.190 |  |
